# Multimodal imaging of optic nerve head abnormalities in high myopia

**DOI:** 10.3389/fneur.2024.1366593

**Published:** 2024-04-23

**Authors:** Ruihan Hu, Qiuyan Wu, Zuohuizi Yi, Changzheng Chen

**Affiliations:** Eye Center, Renmin Hospital of Wuhan University, Wuhan, China

**Keywords:** multimodal imaging, high myopia, optical coherence tomography, optic neuropathy, optic disk

## Abstract

Highly myopic optic nerve head (ONH) abnormalities encompass a series of complications resulting from the stretching of papillary and peripapillary structures during significant axial elongation. The morphological changes in the ONH typically initiate with disk tilting or rotation, progressing to PHOMS and PPA. Tissue defects in each layer manifest as focal lamina cribrosa defects (FLDs), peripapillary intrachoroidal cavitations (PICCs), and acquired pits of the optic nerve (APON). Anterior vitreous/vascular traction and posterior scleral protrusion may lead to prelaminar schisis as well as paravascular cysts and holes, which can potentially develop into retinoschisis. Traditional color fundus photography (CFP) is often insufficient for visualizing most of these lesions, yet their description and quantification benefit significantly from the advancements in optical coherence tomography (OCT) and OCT angiography (OCTA), complemented by fundus autofluorescence (FAF), indocyanine green angiography (ICGA), and three-dimensional imaging. The effective diagnosis and classification of ONH abnormalities heavily rely on a comprehensive understanding of their multimodal imaging features, as outlined in this review. These findings provide valuable insights into optic neuropathy in high myopia, establishing a solid foundation for future endeavors in disease monitoring and treatment guidance.

## 1 Introduction

Myopia is an escalating global public health concern, with projections indicating a rise in prevalence from 22.9% (2 billion) to 49.8% (4.76 billion) by 2050. Approximately 938 million individuals, constituting 9.8% of the global population, are affected by high myopia, typically characterized by a refractive error ≤ −6 diopters (D) (spherical equivalent) or an axial length (AL) ≥26.5 mm (1, 2). Notably, the prevalence of myopia and high myopia is higher in East Asians compared to non-Asian populations. Pathologic myopia accounts for 12–27% of cases of low vision in Chinese and Japanese populations. Furthermore, population-based studies and hospital investigations have identified a significantly elevated incidence of optic nerve damage in highly myopic individuals. Like macular degeneration, choroidal neovascularization, and retinal detachment, optic neuropathy, a common complication of high myopia, can lead to severe visual impairment, reduced quality of life, and associated psychosocial difficulties.

Myopic fundus commonly exhibits pronounced thinning of the peripapillary retinal, choroidal, and scleral layers, along with a temporal/inferior displacement of Bruch’s membrane opening (BMO). Highly myopic eyes may exhibit additional changes, including enlargement of all layers of the optic nerve canal, elongation and thinning of the lamina cribrosa (LC), and the development of circular parapapillary beta, gamma, and delta zones (3). These structural changes give rise to various types of optic nerve head (ONH) abnormalities, recently categorized by Jiang et al. into three main groups: optic disk morphologic abnormalities, papillary/peripapillary tissue defects, and papillary/peripapillary schisis (4).

Nevertheless, conventional ophthalmoscopy is inadequate for visualizing the numerous aforementioned abnormalities. Rapid advancements in diagnostic imaging technology have rendered each imaging modality invaluable in providing unique insights. Thus, a comprehensive grasp of multimodal imaging features, as outlined in this article, greatly aids in the effective diagnosis and management of this disease. Rapid advancements in diagnostic imaging technology have rendered each imaging modality invaluable in providing unique insights. Thus, a comprehensive grasp of multimodal imaging features, as outlined in this article, greatly aids in the effective diagnosis and management of this disease (Table 1).

**Table 1:** List of the acronyms of different imaging modalities.

Acronym	Definition
CFP	Color fundus photography
IR	Infrared reflectance
NIR	Near-infrared reflectance
RFP	Red-free photography
MCI	Multi-color imaging
OCT	Optical coherence tomography
OCTA	Optical coherence tomography angiography
FAF	Fundus autofluorescence
FFA	Fundus fluorescein angiography
ICGA	Indocyanine green angiography

## 2 Optic disk morphologic abnormality

### 2.1 Optic disk tilt and torsion/rotation

Optic disk tilt and optic disk torsion, two prevalent morphological changes observed in myopic eyes, have been increasingly differentiated by researchers (5). Both changes are believed to arise from scleral stretching of peripapillary regions and exhibit a positive correlation with the severity of myopia. Optic disk tilt is characterized by an oblique orientation of the vertical axis, typically oriented inferonasally, accompanied by elevation of the superotemporal neuroretinal rim. In contrast, optic disk torsion entails the rotation of the optic disk around the sagittal axis, potentially resulting in twisting of the optic nerve (6).

#### 2.1.1 CFP/RFP

Fundus photography enables the detection and measurement of optic disk tilt and rotation. The tilted optic disk presents as vertically oval-shaped and is typically quantified using the ovality index (OI), calculated as the ratio between the longest and shortest diameters of the optic disk. According to the observation of Samarawickrama et al. (7), tilted optic disks have smaller disk diameters, cup diameters, and vertically and horizontally oriented cup-to-disk ratios (CDRs), which is consistent with Tong L’s finding using stereo fundus photographs (8). Rotation is measured between the long axis and the vertical meridian, identified as a vertical line 90°from the horizontal line connecting the fovea and the center of ONH. In non-glaucomatous eyes, superior rotation is more prevalent than inferior rotation, with the latter positively correlated with intraocular pressure (IOP), AL, and the area of the beta-zone peripapillary atrophy (PPA) (5). Based on deep learning, Cho et al. developed an automated system for detecting optic disk tilt, demonstrating outstanding agreement with established clinical criteria (9). Optic disk tilt or torsion results in a redistribution of retinal nerve fiber layer (RNFL) thickness, manifested as decreased RNFL visibility on red-free fundus photography (RFP) (10).

#### 2.1.2 OCT

**Figure 1:**
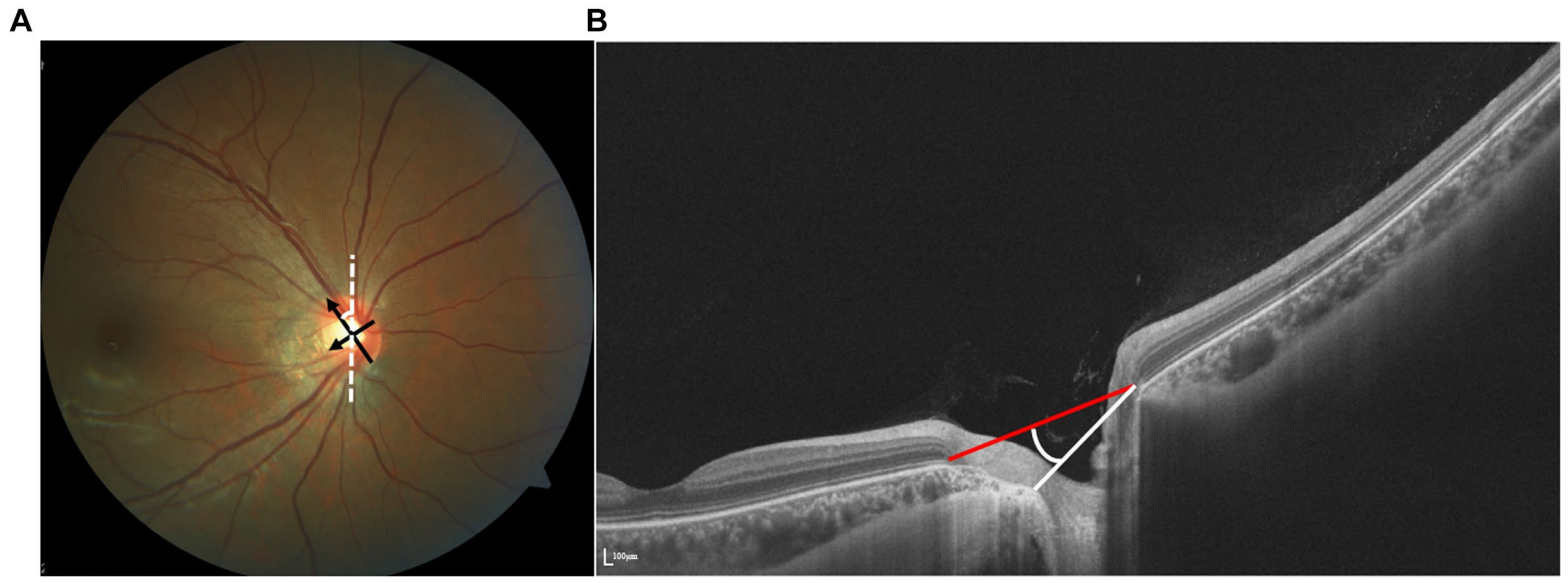
**(A)** Identification of tilt ratio and torsion degree. Tilt ratio was defined as the ratio between the longest diameter and the shortest diameter of the optic disk. Torsion degree was measured between the longest diameter and a vertical line 90° from the horizontal line connecting the fovea and the disk. **(B)** The vertical tilt angle is defined as the angle between the reference plane and the clinical ONH margin plane on the OCT image.

Both the OI and rotation angle, derived from flat projection measurements, are susceptible to observer eye position and thus subject to variability. Optical coherence tomography (OCT), an emerging technology for high-resolution cross-sectional imaging, offers more precise measurements by replacing the optic disk edge with BMO and facilitating quantification (Figure 1). Leung et al. utilized Cirrus SD-OCT to measure the angle between the superotemporal and inferotemporal RNFL bundles, noting a reduction in angle magnitude with increasing AL (11). Since the optic disk shape is determined by the configuration of the neural canal opening on OCT imaging, Jonas interpreted the oblique orientation of the ONH and the oval shape of the optic disk as outcomes of BMO shifting, suggesting a correlation between disk tilt and other morphological alterations (12).

### 2.2 Peripapillary atrophy

PPA encompasses a range of morphological abnormalities resulting from varying degrees of extension, displacement, and atrophy of chorioretinal layers during axial elongation in high myopia. Initially observed as a crescent shape under ophthalmoscopy, PPA has been referred to in the literature as myopic crescent, temporal crescent, sickle, or conus myopicus (13). This region can be subdivided into four zones: peripapillary alpha, beta, gamma, and delta zones (14). Multimodal imaging features of the four zones are shown in Table 2.

**Table 2:** Imaging and histological manifestations of peripapillary alpha, beta, gamma, and delta zone.

Peripapillary zones	Histological definition	Location	Features on fundus images	Features on OCT images	Features on angiography images
Alpha	The presence of BM and RPE, with the latter being irregularly structured	The most peripheral; most frequently located in the temporal horizontal sector	The largest zone characterized by irregular hyper and hypopigmentation	An irregular retinal pigment epithelium (RPE) with the presence of a Bruch’s membrane (BM)	No specific performance
Beta	The presence of BM and absence of RPE, followed by a loss of photoreceptors and finally a closure of the choriocapillaris	Closest to the optic disk border; largest and most frequently located in the temporal horizontal sector	Showing visible sclera as well as large choroidal vessels	Absence of the RPE and the presence of BM	Appears as a hypofluorescent area in angiography; a complete localized microvasculature dropout in OCTA
Gamma	The absence of BM, RPE, choriocapillaris, and deep retinal layers	Between the beta zone (or alpha zone in the case of an absence of beta zone) on its peripheral side and the peripapillary ring on its central side; most frequently in the temporal sector, but can be circular in highly myopic eyes	Whitish area without underlying choriocapillaris, middle-sized choroidal arteries, and signs of RPE	Absence of both the RPE and BM with the presence of only the peripapillary RNFL	Visualizes only the superficial peripapillary retinal capillary system in OCTA
Delta	Elongation and thinning of the peripapillary scleral flange	In the central part (in direction of the optic disk) of the gamma zone	Delineated from the remaining peripheral gamma zone by a demarcation line close to the peripapillary arterial circle Zinn-Haller; often appears darker than the gamma zone	Elongated areas between the openings of the choroidal and the scleral flange	Absence of microvessels larger than 50 microns usually within the gamma zone

#### 2.2.1 CFP/IR

PPA represents one of the earliest clinically observed fundus signs in progressive myopia, exhibiting varying colors due to structural misalignment of the disk margin and variations in pigmentation. The outer boundary of PPA was delineated as the visible scleral edge in color photographs, aligning with the termination of the hyperreflective region in infrared (IR) images. Based on size and morphological features, a succinct classification scheme, including temporal PPA (confined to the temporal aspect of the optic disk), advanced PPA (extending to both superior and inferior aspects of the optic disk), and annular PPA (encompassing the entire peripapillary region), is introduced to intuitively indicate the severity of myopia (15). Lu et al. developed a computer-aided measuring tool, enabling the quantification of PPA size on two-dimensional fundus images, marking the first instance of such quantification (16). Sharma et al. proposed a unified model combining deep neural networks and statistical techniques, which demonstrated excellent accuracy and specificity for automated PPA detection in retinal images (17).

#### 2.2.2 En face/OCT

**Figure 2:**
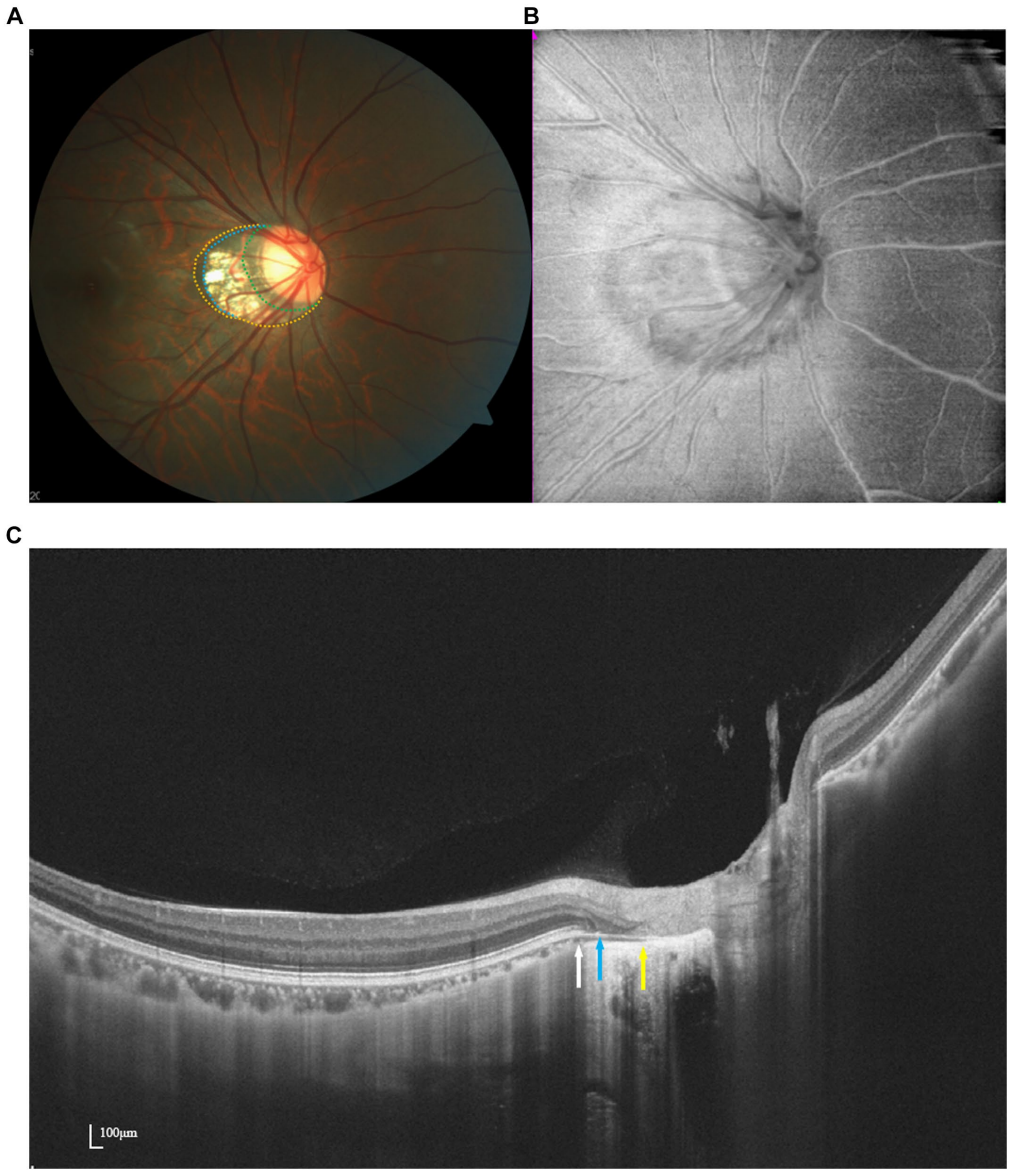
Multimodal imaging of PPA. Optic disk photograph **(A)** and en face image **(B)** show a myopic crescent temporally. Peripapillary alpha zone (yellow dots) appears as a hyperpigmentation zone. Peripapillary beta zone and gamma zone (blue dots) are characterized by a whitish area at the temporal optic disk border with underlying visible sclera and choroidal vessels. Delta zone (green dots) appears darker than the surrounding gamma zone. OCT imaging **(C)** demonstrates the borders of beta, gamma, and delta zone at the ends of RPE (white arrow), BMO (blue arrow), and the end of the scleral flange (yellow arrow).

Delimiting the borders of PPA on fundus images can pose challenges, particularly in eyes with optic disk distortion. Using en face images generated from SS-OCT three-dimensional data, Atsuya Miki succeeded in effectively identifying PPA zones, even in the presence of distorted disk margins (18) (Figure 2). Their investigation revealed an association between the beta zone and glaucoma, as well as age, whereas the gamma zone exhibited a correlation with myopia but not with glaucoma. Despite the typical association between PPA area size and AL, studies by Chen et al. and Hu et al. using SS-OCT images indicated a weak correlation between PPA area size and RNFL thickness (19, 20).

The introduction of OCT has provided fresh insights into PPA. According to Saito, the beta zone corresponds to canal retinal pigment epithelium (RPE) atrophy, while the gamma zone is characterized by an externally oblique configuration of the choroidal border tissue (21). In SS-OCT images, the gamma zone serves as an indicator of the extent of misalignment between the BMO and the anterior scleral canal opening (ASCO), showing a stronger correlation with AL compared to other deep ONH parameters.

#### 2.2.3 OCTA/ICGA

OCTA enables the exploration of choroidal microcirculation features within PPA. Tang et al. observed a reduction in peripapillary vessel density (PVD) in patients’ eyes, notably in the superior nasal (SN), nasal superior (NS), nasal inferior (NI), inferior temporal (IT), temporal inferior (TI), and superior temporal (ST) directions, accompanied by a significant expansion of PPA area (15). The OCTA choroid map delineated a localized microvasculature dropout (MvD) within the beta zone-PPA region, consistent with hypofluorescence observed in indocyanine green angiography (ICGA) (22). Hu et al. identified topographic disparities in radial peripapillary capillaries (RPCs) and choroidal microvasculature across PPA subzones. Both gamma and beta zones exhibited notable reductions in choriocapillaris density compared to the alpha zone, with widths showing negative correlations (23). Additionally, this altered perfusion status coincided with central scotoma, suggesting potential vascular involvement in the progression of visual field (VF) loss and implicating vascular aspects in glaucomatous damage.

### 2.3 Peripapillary hyperreflective ovoid mass-like structures

Peripapillary hyperreflective ovoid mass-like structures (PHOMSs), rather than being solely considered a precursor or subtype of optic disk drusen (ODD), have been distinctly characterized as indicators of axoplasmic stasis on OCT (24). It is hypothesized that in a myopic tilted disk, distended axons herniated into the peripapillary retina may be wedged between the peripapillary nerve fiber layer and Bruch’s membrane, forming a torus or doughnut-like structure around the disk margin, which are identified as PHOMS (25–27).

#### 2.3.1 CFP/IR/NIR/FAF

A PHOMS presents as an elevated and blurred disk margin in fundus photography, while its exact boundary appears as a hyporeflective ring nasal to the disk margin on IR or near-infrared reflectance (NIR) (28). However, other causes of disk swelling, such as papilledema, may also exhibit similar features, leading to clinical misdiagnoses (29).

Differential diagnosis can be facilitated through fundus autofluorescence (FAF), where PHOMS typically show no autofluorescence except for occasional small hyperautofluorescent spots, distinguishing them from ODDs (28, 30).

#### 2.3.2 OCT/ultrasound

As suggested by their name, typical PHOMS present ovoid atop BM with a diffusely hyperreflective signal on OCT, resembling the reflectivity of the RNFL, and lacking complex geometric shapes or signs of intruding surrounding tissues (31). EDI-OCT, which plays a central role in diagnosis, reveals a distinctive feature wherein hyporeflective borders surround hyperreflective structures (30).

In ultrasonography, Mezad et al. identified a novel feature of PHOMS as small hyperechoic structures without posterior shadowing. Furthermore, ultrasound aids in distinguishing the coexistence of PHOMS with optic nerve head drusen (ONHD), indicated by significant posterior shadowing, or with suspicious papilledema, indicated by excessive optic nerve sheath fluid (28).

#### 2.3.3 OCTA/FFA

Fluorescein angiography (FA) may not be an optimal method for identifying PHOMS, as it merely reveals a hyperreflective optic disk with an indistinct boundary (30).

**Figure 3:**
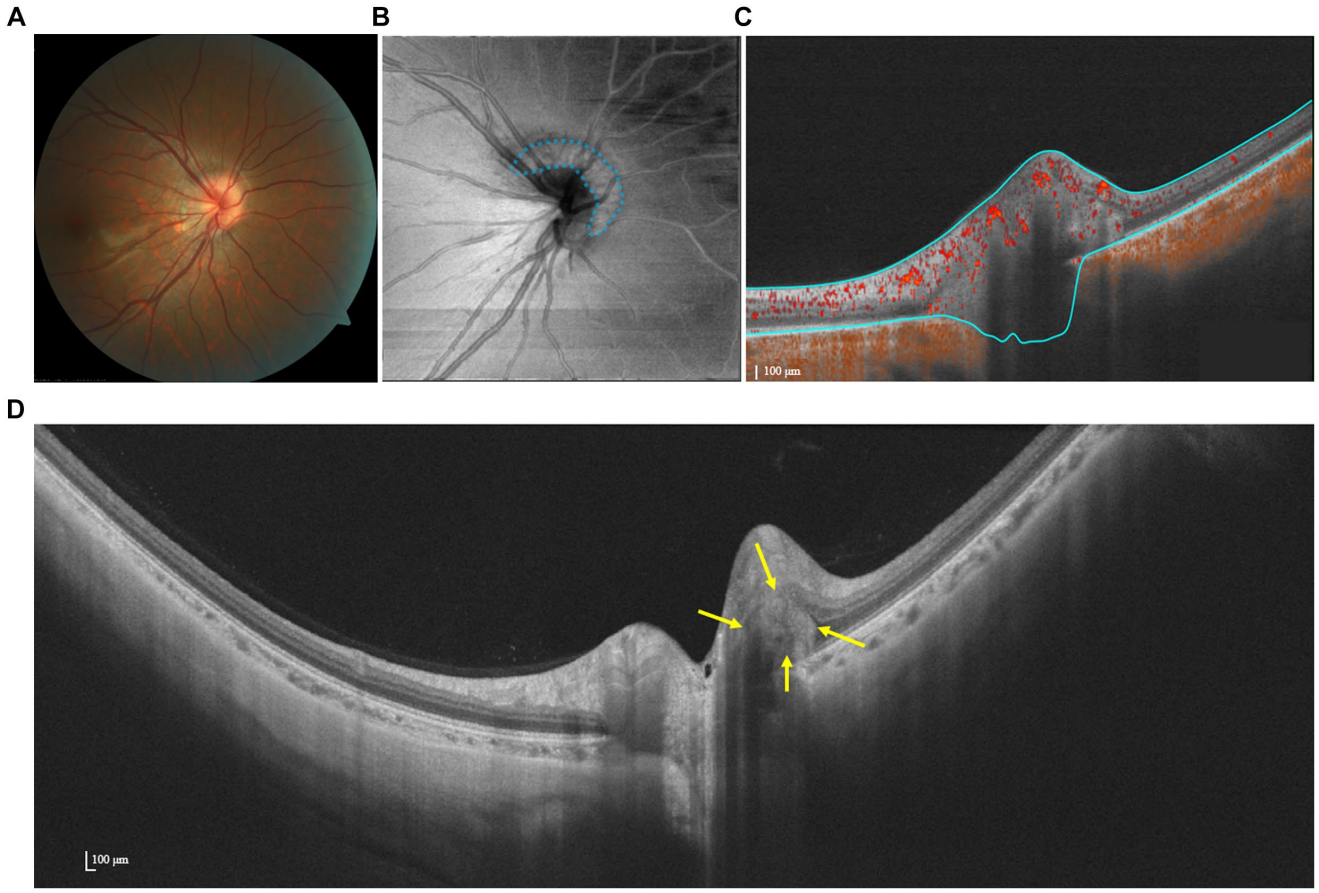
Multimodal imaging of PHOMS. **(A)** Color fundus photography shows a blurred and elevated disk margin. **(B)** The en face image indicates the presence of a hyper-reflective structure (PHOMS) that surrounds the disk, as marked by blue dots. **(C)** OCTA shows a flow signal within the hyperreflective. **(D)** SS-OCT image indicates that the lesion is wedged between Bruch’s membrane and the RNFL.

OCTA demonstrates a flow signal within the hyperreflective lesion, suggesting an intrinsic vascular supply (Figure 3) (32). This hypothesis was subsequently supported in an OCTA-based observation, wherein the presence of a vascular complex within the hyperreflective structure was detected. Additionally, OCTA facilitated the identification of the potential progression of PHOMS into choroidal neovascularization (32). Meanwhile, as reported by Ahn et al., papillary and peripapillary vessel densities decreased significantly in eyes harboring large PHOMS (height ≥ 500 μm) compared to controls (33). Further studies on quantification and determining the permeability of vascular structures in and around PHOMS are warranted.

## 3 Papillary/peripapillary tissue defect

### 3.1 Focal lamina cribrosa defects (LCDs)

In highly myopic eyes, the LC undergoes stretching-associated morphological alterations, characterized by significant thinning, displacement, and curvature. Additionally, there is an augmented trans-LC pressure gradient, predisposing individuals to a heightened risk of developing focal lamina cribrosa defects (LCDs) (12). Furthermore, numerous researchers have verified the association between focal LC defects and localized RNFL, or neuroretinal rim loss, sometimes presenting clinically as acquired pits of the optic nerve (APON).

#### 3.1.1 CFP

Color fundus photography (CFP) may not consistently reveal an obvious LCD; however, occasionally, a dark yellowish-orange lesion can be discerned in the area of the myopic disk. Utilizing fundus stereophotographs, Tatham et al. confirmed the correlation between LCDs and localized RNFL defects in glaucomatous eyes (34).

#### 3.1.2 En face/OCT

Recent advancements in OCT imaging modalities have facilitated *in vivo* imaging of the LC. En face volume images offer improved visualization of LC defects, identified as hyporeflective spots amidst the highly reflective LC (35). In SS-OCT images, a focal LC defect is characterized as a loss of high reflectivity from the anterior–posterior border of the full-thickness LC in the vertical, horizontal, and axial serial B-scan images. Originally, Kiumehr et al. delineated five categories of LCDs based on their shape: smooth indentation, moth-eaten appearance defect, step-like depression, hole-like defect, and altered laminar insertion (35). Similarly, after comparing 3D SS-OCT images with en face images, Takayama et al. classified LC defects into lamina cavity and lamina disinsertion, both exhibiting irregular and full-thickness loss of lamina reflectivity, distinguishable from artifacts attributed to vascular shadowing (36). EDI-OCT further improved the visualization of the whole structure, particularly beneath the neuroretinal rim. Based on EDI-OCT, Han et al. proposed another classification, distinguishing between lamina holes, indicating localized discontinuities, and LC disinsertion type, denoting the detachment of the peripheral LC and a downward slope toward the neural canal wall. They additionally noted that the latter were predominantly situated in the gamma zone and displayed a stronger correlation with glaucoma and parameters associated with myopia (37).

The entire shape of LC defects was still hindered by optic disk tilts and rotations in highly myopic eyes until recently. Ota-Itadani et al. developed a deep convolutional neural network (DCNN) trained on manually labeled cross-sectional OCT data extracted from 256, 222 3D OCT images to automatically segment LC defects and generate three-dimensional images. Through this method, they initially identified common locations of LC defects in stereo space (38). The application of such novel methodologies may also aid in identifying regions of structural weakness that may predispose individuals to diseases.

#### 3.1.3 OCTA

In glaucomatous eyes, histological and OCTA-based studies have unveiled a strong topographic correlation between focal LC defects and dropout of parapapillary capillaries (39–41). Lee et al. further confirmed that glaucomatous LC defects were more likely to originate from circulation loss than mechanical strain (42). However, this conclusion might not be applicable to highly myopic eyes, as researchers excluded eyes with myopic tilted disks and gamma zones. Choe et al. initially demonstrated a significant reduction in PVD or perfusion density (PD) using OCTA with the development of focal LC defects in young myopic patients, suggesting an increasing risk of glaucoma in the future (43).

### 3.2 Peripapillary intrachoroidal cavitation

PICC, initially proposed as peripapillary detachment in pathologic myopia (PDPM) by Freund et al., was later renamed by Toranzo et al. after its intrachoroidal location was revealed (44, 45). While PICC can occur in non-highly myopic and even non-myopic eyes, it is more prevalent in highly myopic eyes, particularly those with other myopic complications such as posterior staphyloma, tilted disk, and a higher maculopathy category (46, 47).

#### 3.2.1 CFP/IR/MCI

A PICC can manifest as a well-circumscribed, yellow-orange lesion along the border of the optic disk on fundus photography or as a peripapillary dark reflective region on IR reflectance. It is noteworthy that while most lesions are positioned adjacent to the inferior edge of the optic disk, other sections and even the entire peripapillary circular region may be affected. However, such appearances are evident in only 46.7–53% of PICCs detected via OCT.

Multi-color images (MCIs) of PICC illustrate a well-circumscribed, caesious lesion contiguous with the ONH. Despite their advantages over fundus color photographs in detecting “pits” in retinal layers, MCIs might struggle to identify PICCs due to their greater depth and the absence of melanin within the choroidal cavitation (47, 48).

#### 3.2.2 OCT

**Figure 4:**
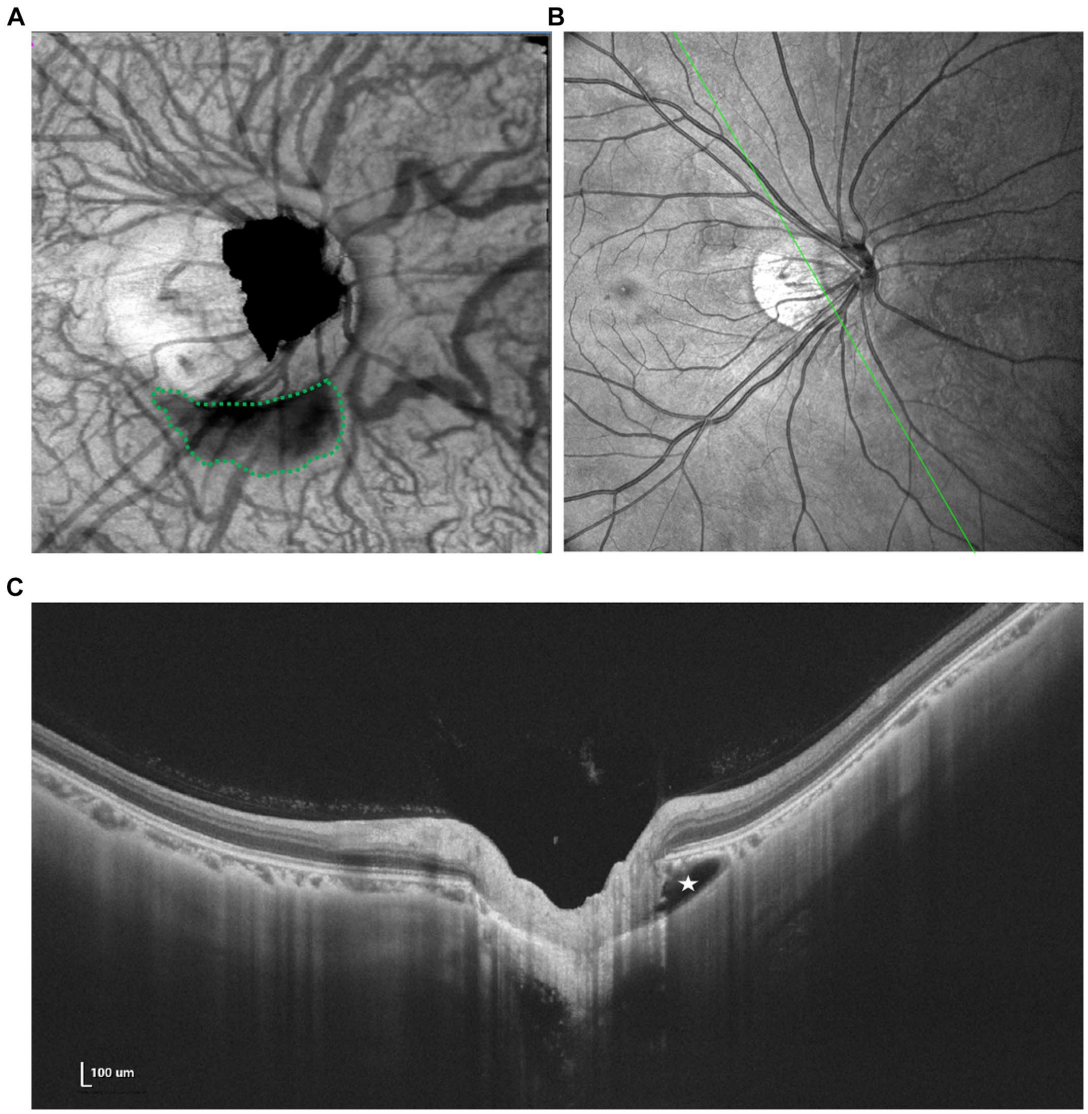
Multimodal imaging of PICC. **(A)** En face image shows an irregular hyporeflective area (green dots) inferior to the optic disk. **(B,C)** Spectral domain OCT revealed a large intrachoroidal hyporeflective space below the normal RPE plane within the PICC (white star).

Based on OCT manifestations, PICC is characterized as a hyporeflective triangular thickening of the choroid with the base at the optic disk border in horizontal or vertical sections, occasionally displaying discontinuity of the normal overlying RPE (49, 50) (Figure 4).

Similar OCT-morphologic changes have been observed at the border of colobomas, which was considered a different stage of the same disorder as PICC (51). The utilization of SS-OCT provides more detailed imaging that may elucidate the correlation between PICC and other complications as well as visual function. Ehongo et al. described a shared anteroposterior alignment among expanded subarachnoid space (SAS), exclusive staphyloma-related choroidal excavation (SCD), and the wedge configuration of the posterior choroidal wall, along with the discontinuity of the embedded ONH border tissue and additional choroidal thickness coinciding with dural traction. They hypothesized that PICC is actually a type of suprachoroidal detachment, following the collapse of the scleral flange caused by the tensile forces of the optic nerve sheaths during adduction (52). Based on SS-OCT, Xie et al. assessed the correspondence between structural characteristics and VF defects, highlighting that instead of the presence of PICC itself, herniation and eventual loss of local retinal tissue may be the true reason behind VF defects (30).

Efforts have also been made to obtain the three-dimensional or volume parameters of PICC. Rohan et al. achieved a side profile view of PICC through three-dimensional disk cube reconstruction imaging. Additionally, Fujimoto et al. successfully implemented a deep learning-based denoising, delineation, and 3D rendering system to establish PICC volume as a new three-dimensional parameter (53, 54). They subsequently demonstrated its ability to reflect the correlation between PICC volume and VF sensitivity.

#### 3.2.3 OCTA/FFA/ICGA

The vascular characteristics of PICC are accessible through multimodal angiography.

FA reveals early hypofluorescence of the lesion followed by progressive staining without any dye pooling, which can be attributed to the disorganization of choroidal architecture and subsequent scleral impregnation. This helps differentiate PlCC from pigment epithelial detachment, peripapillary choroidal neovascularization, and other potentially confounding entities. Meanwhile, ICGA demonstrates absent choroidal flow, resulting in hypofluorescence throughout the sequence (55).

In highly myopic eyes with PICC, OCTA has revealed a significant reduction in vessel density not only at the choroidal level but also at the level of the RPC. Based on B-scans of OCTA, Kim et al. observed a preserved choriocapillary signal against Bruch’s membrane, which completely vanished within the hyperreflective cavity (56). Another notable vascular abnormality involves the TI vein, which has been described in several cases as either submerging into the intrachoroidal cavitation (ICC) or covering the sinkhole of the PICC (48, 57). The advancement of OCTA technology allows for the early recognition of subtle losses in the microcirculatory network in PICC, facilitating a detailed exploration of its pathophysiological mechanisms.

### 3.3 Acquired pits of optic nerve

Pits in the optic disk area have been generally understood as congenital, although they may also develop in advanced glaucoma. With the evolution of imaging modalities, particularly OCT, our understanding of this disorder of high myopia continues to expand. It is presumed that myopic APON constitute an independent entity distinct from glaucoma-related pits. This differentiation is based on the characteristic development of a glaucomatous pit, which typically arises from the progressive thinning and bulging of the LC—an area that is stretched and taut in highly myopic eyes (12).

#### 3.3.1 CFP

The presence of a myopic APON is scarcely discernible with CFP. A review of medical records from 198 myopia patients revealed that only 2 out of 32 cases of peripapillary pits were successfully observed as yellowish-gray to dark circular spots, with color variation depending on the quantity and distribution of glial tissue within the pit (58).

#### 3.3.2 En face/OCT

En face views of the optic disk area presented the triangular hyporeflective opening of most pits, with the base at the outer edge of the optic disk and the apex heading into the center of the disk. Utilizing en face images from vertical and horizontal SS-OCT scans, Ohno-Matsui et al. identified pits in 16.2% of highly myopic eyes (mean AXL: 30 ± 2 mm), categorizing them into optic disk pits (located in the optic disk area) and conus pits (located in the myopic conus outside the optic disk) (58).

A definitive diagnosis of myopic APON requires OCT imaging, which reveals a deep excavation localized in the superior/inferior border of the optic disk (optic disk pits) or the nasal/temporal side of the scleral ridge (conus pits). This excavation is accompanied by discontinuous laminar architecture and overlying nerve fiber tissues. Moreover, there is little or no rim tissue remaining adjacent to the pit, which manifests as a hyporeflective gap at the junction of the LC and the peripapillary sclera (58). The accompanying structural characteristics of the pits vary, ranging from the dehiscence of the sclera to an attached ICC (59).

#### 3.3.3 OCTA

Angiography plays a pivotal role in imaging pits. Using SS-OCT combined with fluorescein and ICGA, Ohno-Matsui et al. demonstrated that two conus pits took the form of an enlargement of the emissary canals for the short posterior ciliary arteries (SPCAs) (58). These findings align with the hypothesis that mechanical expansion of the papillary and peripapillary regions may be the primary cause of the pits in these highly myopic eyes. Kita et al. separately observed localized retinal nerve fiber bundle and perfusion damages based on circumpapillary retinal nerve fiber layer (cpRNFL) OCT and OCTA, respectively. These damages spatially corresponded to the pit area and, more importantly, to a progressing visual field defect (VFD) (paracentral scotoma) (60).

## 4 Papillary/peripapillary schisis

### 4.1 Paravascular abnormalities (PVAs)

Various types of abnormalities have been reported to manifest at or around retinal vessels in highly myopic eyes, including paravascular retinal cysts, paravascular microfolds, and paravascular lamellar holes. These concepts may partially overlap with paravascular inner retinal defects (PIRDs) proposed by Muraoka et al. (61). While several isolated case reports have identified paravascular retinal rarefaction and microholes around posterior staphyloma III and IX (62) there has been no specific study describing PVAs in the papillary or peripapillary areas to date. Nevertheless, the occurrence of papillary/peripapillary PVAs is likely given the optic disk’s richness in major retinal vessels and their relevant interactions.

#### 4.1.1 CFP/RFP

Generally, PVAs appear as spindle-shaped or caterpillar-shaped dark areas along the major retinal vessels, particularly the temporal vascular arcades, in fundus photographs (63). On red-free images, they manifest as retinal rarefaction, characterized by increased contrast (61).

#### 4.1.2 En face/OCT

**Figure 5:**
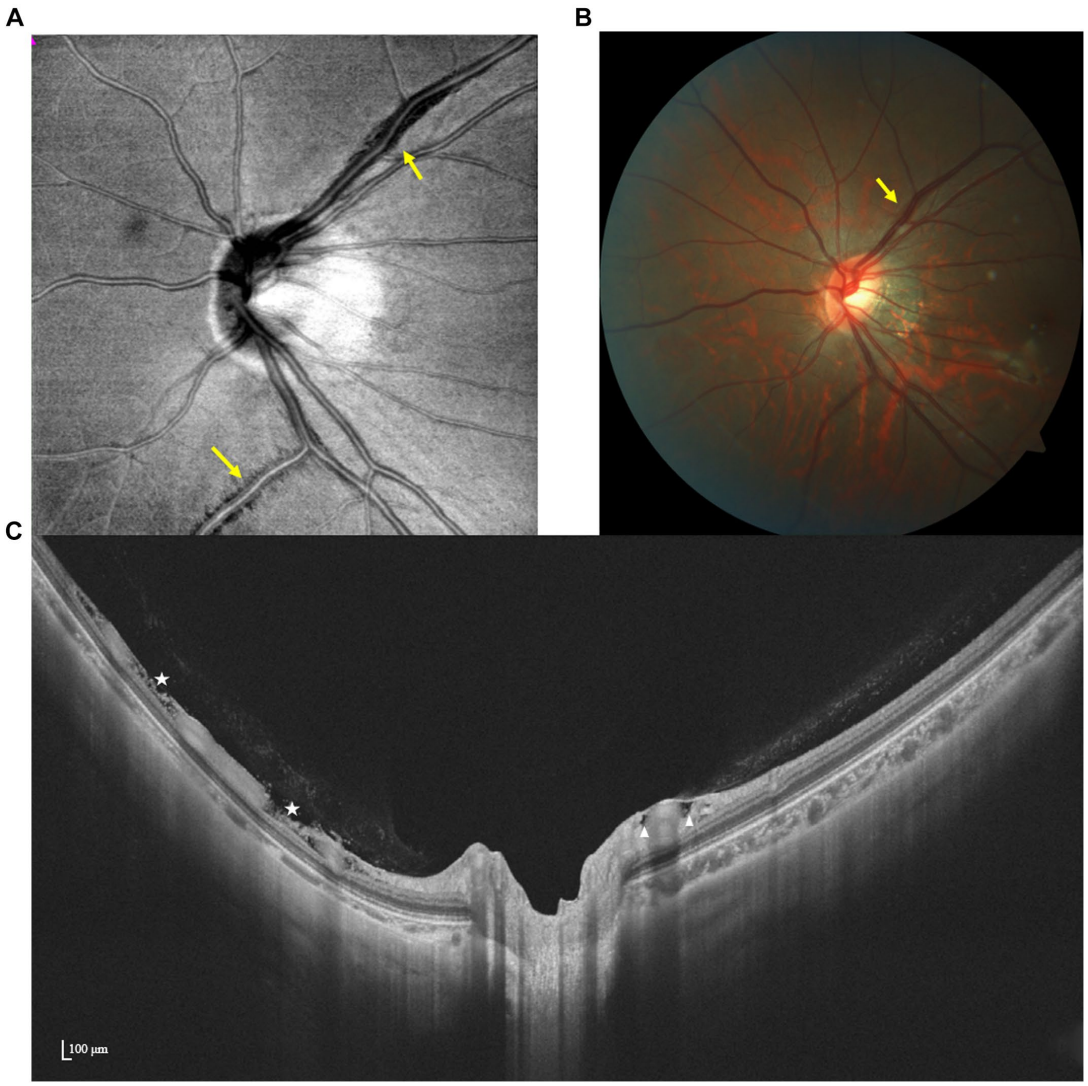
Multimodal imaging of PIRDs. **(A,B)** Dark fissure-like changes (yellow arrow) are observed along the upper temporal retinal vessel on the en face and color fundus image. **(C)** Isolated cystoid structures (white triangles) adjacent to the branch of the upper temporal retinal vessel and fissure-like structures (white stars) adjacent to the branch of the lower temporal retinal vessel are manifested by SS-OCT.

Using OCT, three distinct features can be observed in the paravascular region of the retina: microfolds, cysts, and lamellar holes. Microfolds appear as projections from retinal vessels into the vitreous cavity, cysts appear as hyporeflective spaces surrounding the vessels, and lamellar holes appear as discontinuities extending from the inner limiting membrane to between one-half and two-thirds the thickness of the neural retina, all adjacent to retinal vessels (64, 65) (Figure 5). Since PVAs may constitute the anatomic basis of retinoschisis and even detachment, timely identification and treatment become crucial (66, 67).

SS-OCTA en face structural images identified dark, scalloped regions along major retinal veins that match in location with PVAs on B-scans (68). Since PVAs can extend beyond the macula and optic disk areas, using wide-field en face imaging simplifies capturing PVAs across a wider range in a single shot, thereby enhancing efficiency in the process (61).

### 4.2 Peripapillary retinoschisis (PPRS)

Myopic retinoschisis, characterized by splitting of the retina, typically between the macula and optic disk, is recognized as a precursor of myopic traction maculopathy. However, PPRS has been primarily discussed in the context of glaucoma rather than high myopia in previous studies. Based on research by Sherman et al. and Li et al., the incidence of PPRS in highly myopic eyes increases from 3% (19/600) to 27.5% (123/448) with age (69, 70).

#### 4.2.1 CFP/IR/RFP

Generally, PRRS is nearly invisible in color fundus photographs. Nevertheless, traction-related displacement of split tissues may create the appearance of retinal thickening, manifested by a slight elevation of the local retina. This corresponds to the pattern observed in retinal thickness maps, commonly seen as retinal edema extending toward the optic disk (71).

**Figure 6:**
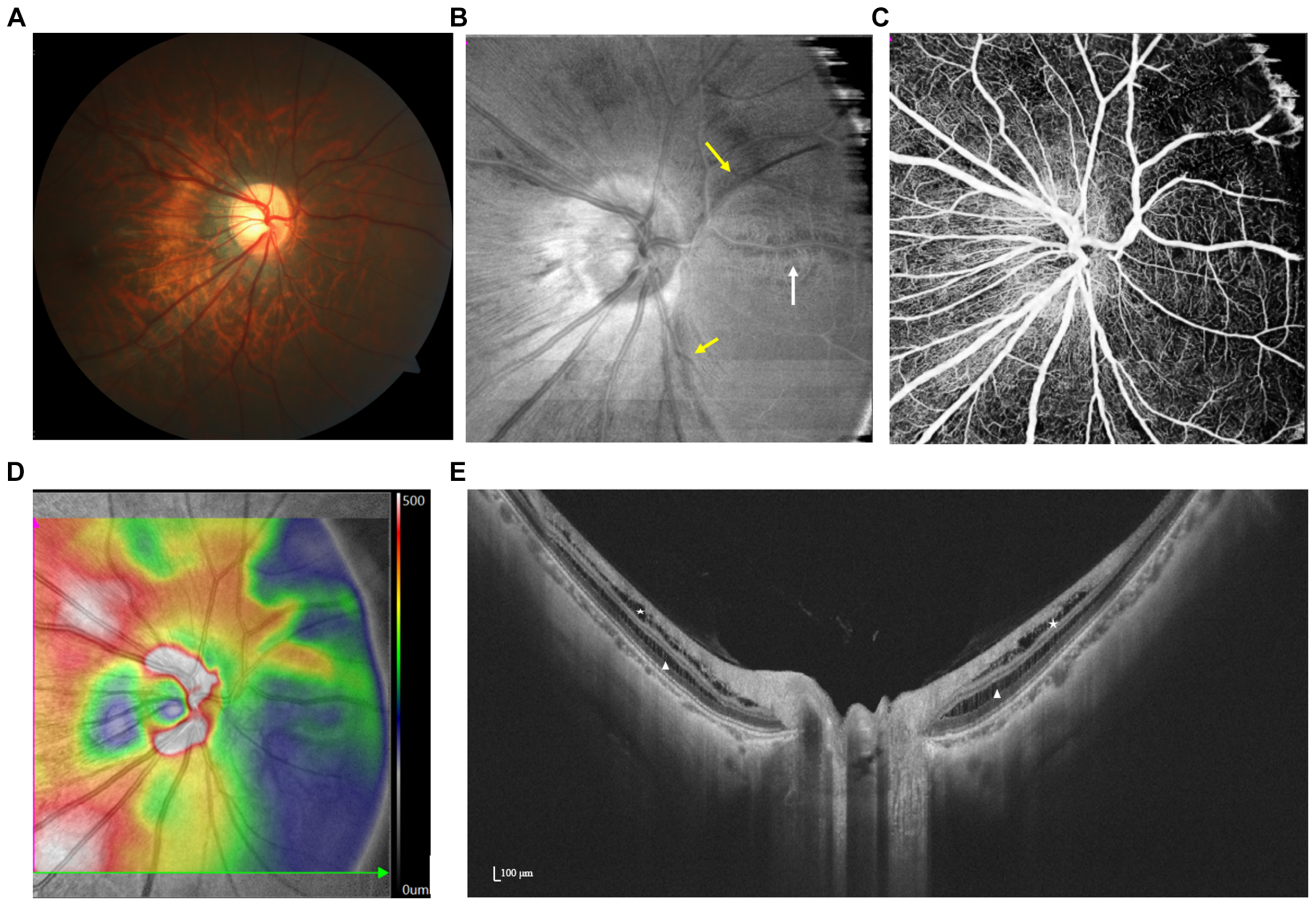
Spectrum of PPRS with various features illustrated by multimodal imaging. **(A,B)** En face image reveals that irregularly hyporeflective areas (yellow arrows) extend along the nerve fibers, which are barely visible by fundus photography. A paravascular schisis can also be seen (white arrow). **(C)** Focal loss of retinal capillaries on OCTA and **(D)** artifactual thickening on the retinal topographic map correspond with the location of the schisis. **(E)** SS-OCT demonstrates peripapillary retinoschisis in both the inner (white triangles) and outer (white stars) layers.

In the IR image, PPRS manifests as a distinct, dark area with a smooth margin, featuring a narrower base connected to the optic disk border and expanding along the path of nerve fiber bundles. Contrarily, the red-free fundus photograph reveals only a localized RNFL defect, whose opening appears like a clear, spindle-like dark area (72, 73) (Figure 6).

#### 4.2.2 En face/OCT

First observed by Sherman et al. in highly myopic eyes using SD-OCT, PRRS was documented as splitting situated variably along the disk margin, involving multiple layers, particularly the inner and outer plexiform layers (70). Using wide-field en face SS-OCT, He et al. mapped the distribution of extrafoveal retinoschisis and delineated them as extending hyporeflective areas along the RNFL with well-circumscribed upper and lower margins. Additionally, they identified a distinct dehiscence within the maculopapillary bundles, suggesting a potential non-glaucomatous neuropathy exclusive to high myopia (74).

It is widely acknowledged that two opposing forces provided by posterior staphyloma and anterior vitreous traction contribute to the formation of macular schisis (75). In addition, paravascular microfolds, cysts, and lamellar holes have been implicated in this mechanism (67). Optic disks are also susceptible to the aforementioned abnormalities during axial elongation, as subsequently confirmed by OCT-based detection of their coexistence with PPRS (69). Nonetheless, little is known about the correlation between paravascular lesions and PRRS yet, calling for more detailed studies.

#### 4.2.3 OCTA/FFA

SS-OCTA and en face SS-OCTA images reveal dark back shadowing in the superficial capillary plexus (SCP) or deep capillary plexus (DCP), depending on whether inner or outer retinal layers are involved, accompanied by a markedly increased reflectivity in the RNFL, where retinoschisis occurs (71). During the late phase of FA, no signs of leakage are observed in the peripapillary regions (76).

### 4.3 Prelaminar schisis

Hitherto, it remains unclear whether retinoschisis and prelaminar schisis share a common origin. While the association between retinoschisis and high myopia is recognized, prelaminar schisis has historically been considered more closely related to glaucoma, particularly in eyes without elongated AL (77). However, in a recent cohort study, Xie et al. identified prelaminar schisis in 30.6% of eyes with pathological myopia, among which 54.5% exhibited coexisting retinoschisis. This prevalence notably surpassed that observed in non-pathological myopic glaucomatous eyes (30).

#### 4.3.1 OCT

**Figure 7:**
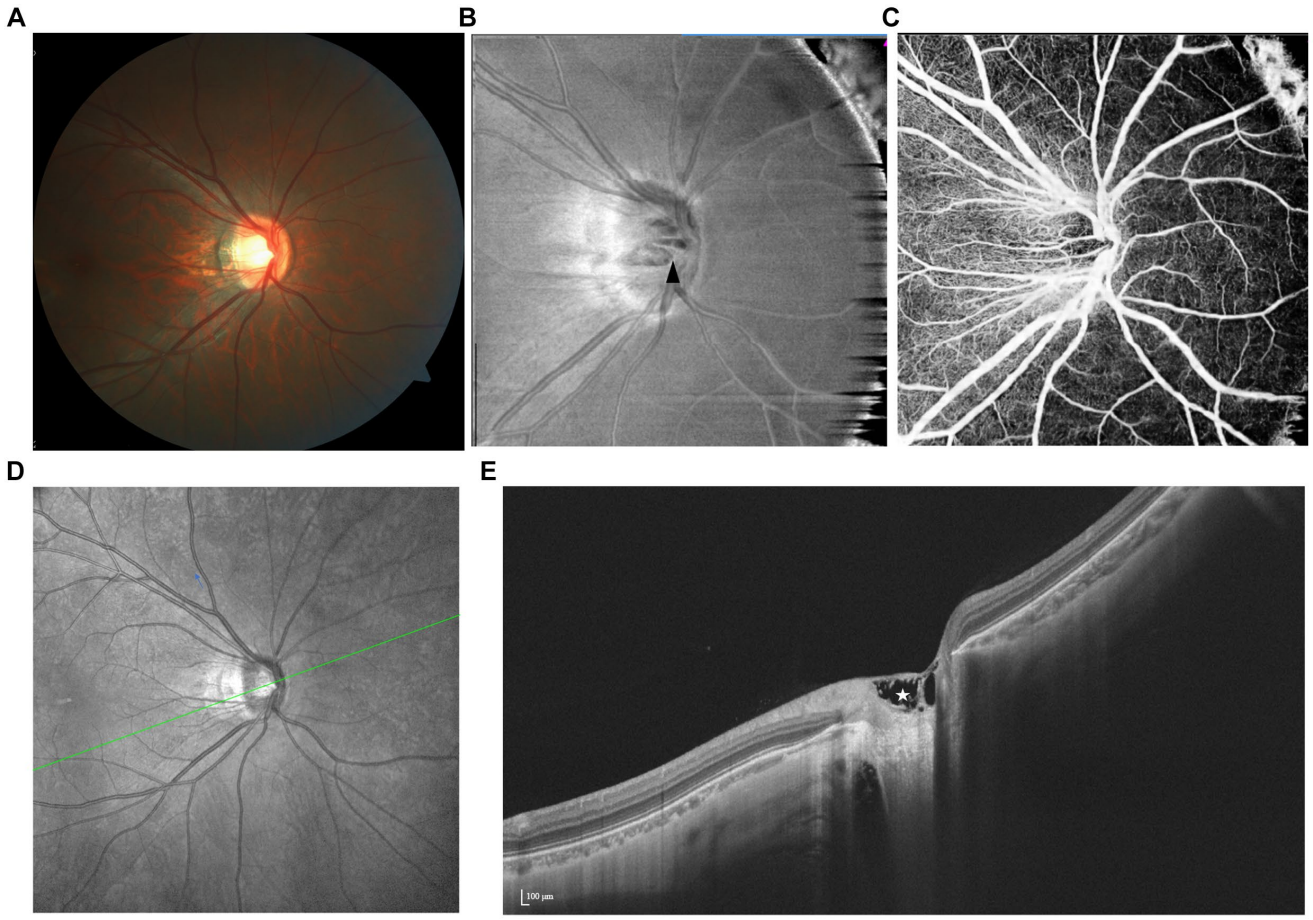
Spectrum of prelaminar schisis with various features illustrated by multimodal imaging. **(A)** The fundus color photograph shows no specific lesion. **(B)** Reflection decreases unevenly within the schisis area (black arrowhead) on the en face image. **(C)** OCTA shows that the location of vascular loss is consistent with the structural change. **(D,E)** On SS-OCT, prelaminar schisis often presents as a splitting of superficial prelaminar tissue.

OCT images usually depict a meshwork-like splitting of the superficial prelaminar tissue in the optic disk, accompanied by a disruption of the prelaminar tissue and an anterior protrusion of retinal vessels (Figure 7). Based on the size and depth of the voids, Lowry et al. categorized the lesions into four levels. They revealed that the presence and severity of these lesions were associated with common OCT parameters such as larger ONH size, thinner neuroretinal rim width, and greater cup depth, indicating a higher risk of ongoing glaucomatous damage (78).

#### 4.3.2 OCTA

As suggested by the OCT images, two initial factors contributing to the development of prelaminar schisis were discussed. Over four-fifths of the lesions are attributed to the stretching and anterior hanging of the retinal vessels, while vitreous or epiretinal proliferation is also considered an underlying cause. Consistent with the first hypothesis, OCTA scanning clearly demonstrated major retinal vessel trunks contained within anteriorly separated tissues in some cases of prelaminar schisis (78). Longitudinal studies investigating structural and vascular changes over time may shed light on the etiology and clinical importance of this abnormality.

## 5 Future prospects

### 5.1 Highly myopic glaucomatous and non-glaucomatous optic neuropathy

Previous studies have demonstrated a correlation between high myopia and an increased prevalence of glaucomatous or glaucoma-like optic neuropathy (GON), particularly pronounced when the AL exceeds 26.5 mm (or -8D). Many of the aforementioned morphological changes have been considered potential contributing factors to this phenomenon. However, clinical detection of GON in highly myopic eyes poses certain challenges, primarily due to the diminished spatial and color contrast between the neuroretinal rim and the optic cup, resulting from the stretching of the LC and a decrease in blood supply. Additionally, assessment of RNFL defects on both fundus photographs and OCT imaging is challenging owing to the bright parapapillary background and the presence of the gamma zone (79). Recent studies have focused on evaluating the neuroretinal rim (including BMO-MRW), the ganglion cell-inner plexiform layer thickness, and the microvasculature of the ONH in order to overcome this difficulty (80–83). Furthermore, efforts to create a novel myopic database are just beginning to emerge.

In cases of high myopia, the development of the temporal parapapillary gamma zone may lead to an increased disk-foveal distance (DFD). Consequent stretching of retinal ganglion cell axons, especially the papillomacular bundle, might occur, potentially elucidating the underlying mechanism (84). Further exploration is needed to confirm whether non-glaucomatous optic neuropathy (NGON) presents as a distinct entity independent from glaucoma.

### 5.2 Structure–function correlation

Elucidating the structure–function correlation of ONH abnormalities in high myopia poses several challenges. First, VFDs caused by macular degeneration and glaucoma are often mistakenly attributed to ONH abnormalities. Ding et al. found that 16.1% of VFDs in young high myopes mimicked classic glaucomatous defects (85). Second, coexisting abnormalities may mutually interfere, resulting in multifactorial causes of VF defects (30). Thirdly, the subjectivity of VF test outcomes heavily depends on patient cooperation. Despite these challenges, Lin et al. addressed a gap by proposing an innovative classification system for non-pathologic eyes, although its real-world clinical performance and validation in population-based screening require further evaluation (86). Prospective efforts toward comprehensively understanding the progression of structural anomalies over time and their corresponding VFDs hold significant promise for informing diagnostic and therapeutic strategies.

### 5.3 Artificial intelligence and myopia management

Timely imaging, coupled with accurate AI-assisted interpretation, is essential for detecting early complications and monitoring myopia progression. While current AI models excel in distinguishing “disease-free” from “diseased” cases in myopia, they face challenges in more complex tasks due to shared pathological features across ophthalmic conditions. Multimodal data fusion techniques integrate relevant features from images for AI processing. For instance, prediction tasks based on CFP images focus on refractive error prediction using ResNet, aiding in both morphological studies of myopic eyes and epidemiological research (87). Additionally, the distinct layer information inherent in OCT images facilitates the segmentation and analysis of choroidal sublayers utilizing methodologies such as U-Net and mask R-CNN (88–90). These features, encompassing geometric measurements and characteristic lesion regions, can even be processed to develop precise models for long-term visual acuity prediction in highly myopic eyes (91, 92). By integrating findings from these modalities, clinicians can tailor treatment plans, monitor disease progression, and optimize visual outcomes for patients with ONH abnormalities in high myopia.

## 6 Conclusion

In summary, ONH abnormalities encompass a variety of specific lesions associated with optic neuropathy in high myopia. Among them, tilted/rotated disks, PPA, and PHOMS are predominantly visible through fundus photography. However, the continuous advancement of imaging technology, particularly OCT, enables definitive diagnosis and further quantitative evaluation with high-resolution images. For lesions accompanied by vascular displacement and MvD, circulation damage is demonstrated through a combination of static OCTA display and dynamic FFA/ICGA imaging. Preliminary establishment of structure–function correlation is feasible in eyes with single abnormalities, where the locations of changes align with patterns of RNFL and/or VF loss. The integration of timely imaging, multimodal data fusion, and AI interpretation is also highly beneficial for precise diagnosis and treatment planning. Given the close correlation between optic neuropathy in high myopia and glaucoma, and the existing deficiency in recognized mechanisms, future research in high myopia necessitates exploration of new biomarkers and prognostic indicators to assess the risk of progression in long-term follow-up.

## Author contributions

RH: Writing – review & editing, Writing – original draft. QW: Writing – original draft, Writing – review & editing. ZY: Writing – review & editing. CC: Writing – review & editing.
