# Supplementary material for: Multimodal imaging of optic nerve head abnormalities in high myopia
Source: Front Neurol. 2024 Apr 23;15:1366593. doi: 10.3389/fneur.2024.1366593 (PMC11075756; doi:10.3389/fneur.2024.1366593)
Supplement: Supplementary file 1 [file Table_1.docx]

Table S1. Features of various ONH abnormalities across different imaging modalities.

|  | **CFP** | **IR/NIR** | **MCI/RFP/FAF** | **En face OCT** | **OCT** | **OCTA** | **ICGA/FFA** |
| --- | --- | --- | --- | --- | --- | --- | --- |
| **Disc tilt/torsion** | vertically oval-shaped/rotated disc | | decreased RNFL visibility(RFP) | BMO shifting | | - | |
| **PPA** | visible scleral edge | hyperreflective region | - | Gray to dark gray aera around the optic disc | atrophy or dislocation in each layer (See table 1) | Various degrees of microcirculation loss(See table 1) | |
| **PHOMS** | an elevated and blurred disc margin | a hyporeflective ring nasal to the disc margin | occasional small hyperautofluorescent spots(FAF) | - | Oval hyperreflective structures atop Bruch's membrane | an intrinsic vascular complex | a hyperreflective and blurred optic disc boundary(FFA) |
| **LCD** | a dark yellowish-orange lesion | - | | hyporeflective spots amidst the highly reflective lamina cribrosa | loss of high reflectivity from the anterior-posterior border of the full-thickness lamina cribrosa | significant reduction in peripapillary vessel density (VD) or perfusion density (PD) |  |
| **PICC** | a well-circumscribed, yellow-orange lesion along the border of the optic disc | a peripapillary dark reflective region | a well-circumscribed, caesious lesion contiguous with the ONH(MCI) | - | Hyporeflective triangular choroidal thickening | Reduced vessel density;preserved choriocapillary signal against Bruch’s membrane;temporal vein involvement | early hypofluorescence, followed by progressive staining without any dye pooling(FFA); hypofluorescence throughout the sequence(ICGA) |
| **APON** | scarcely discernible; yellowish-grey to dark circular spots | - | | triangular hyporeflective pits, with the base at the outer edge of the optic disc and the apex towards the center | a deep excavation accompanied by discontinuous laminar architecture and overlying nerve fiber tissues | localized perfusion damages spatially corresponding to the pit area | - |
| **PVA** | spindle-shaped or caterpillar-shaped dark areas along the major retinal vessels, particularly the temporal vascular arcades | | retinal rarefaction, characterized by increased contrast(RFP) | dark, scalloped regions along major retinal veins | projections from retinal vessels into the vitreous cavity(microfolds); hyporeflective spaces surrounding the vessels(cysts); discontinuities extending from the inner limiting membrane to 1/2~2/3 the thickness of the neural retina(lamellar holes) | - | |
| **PPRS** | nearly invisible; a slight elevation of the local retina | a distinct, dark area with a smooth margin, narrower base connected to the optic disc border and expanding along the path of nerve fiber bundles | localized RNFL defect, whose opening appears like a clear, spindle-like dark area(RFP) | extending hyporeflective areas along the RNFL with well-circumscribed upper and lower margins | splitting situated variably along the disc margin, involving multiple layers, particularly the inner and outer plexiform layers | - | |
| **Prelaminar schisis** | - | | | | Meshwork-like splitting, disrupted prelaminar tissue, anterior vessel protrusion | major retinal vessel trunks contained within anteriorly separated tissues |  |

PPA indicates peripapillary atrophy;PHOMS, peripapillary hyperreflective ovoid mass-like structures;LCD,lamina cribrosa defects;PICC,peripapillary intrachoroidal cavitaion;APON,acquired pits of optic nerve;PVA,paravascular abnormalities;PPRS,peripapillary retinoschisis;CFP,color fundus photography;IR, infrared reflectance;NIR, near-infrared reflectance; RFP, red-free photography; MCI, multi-color imaging; OCT ,optical coherence tomography;OCTA, optical coherence tomography angiography;FAF, fundus autofluorescence;FFA, fundus fluorescein angiography;ICGA, indocyanine green angiography;BMO,Bruch’s membrane opening.
